# Methods for standardising and monitoring surgical interventions in RCTs

**DOI:** 10.1186/1745-6215-16-S2-O34

**Published:** 2015-11-16

**Authors:** Natalie Blencowe, Nicola Mills, Jenny Donovan, Jane Blazeby

**Affiliations:** 1 University of Bristol, Bristol, UK

## Introduction

Multi-centre randomised controlled trials (RCTs) in surgery are challenging. It is particularly difficult to establish standards of surgery and monitor whether interventions are delivered as intended. This can lead to uncertainties about the differences between trial group interventions and the meaning of observed treatment effects.

This study developed and tested methods for intervention design and monitoring within the internal pilot phase of a surgical RCT.

## Methods

Two phases of work were undertaken: i) in-depth analysis of intervention reporting in published surgical RCTs to establish a typology for intervention design, ii) qualitative work in the operating theatre to identify ‘key’ components of interventions, comprising video data capture, non-participant observation of surgical interventions and interviews with surgeons.

## Results

Some 80 RCTs reporting 160 interventions informed the new typology, which provides guidance for describing and monitoring surgical interventions. It prompts trialists to identify components and steps of interventions and consider whether each should be mandated, optional or prohibited within the RCT. The qualitative work identified variations in intervention delivery between surgeons and centres, established key intervention components and steps, and explored surgeons' opinions about how these should be delivered in the trial.

## Conclusions

Ensuring standards of surgery is important in surgical RCTs, even if they are considered to be pragmatic. This study has developed methods for intervention design and monitoring in RCTs. Consensus meetings have been planned with surgeons to agree the final intervention protocol for the main study. These methods are now being tested in the context of other surgical RCTs.

